# Somnambulist on Guard

**Published:** 1875-08

**Authors:** 


A Somnambulist on Guard.—The *Philadelphia Medical Reporter* tells of a soldier at Fort Concho, Texas, who was observed by his lieutenant to walk his rounds in the night without challenging those who approached. His eyes were open, and he made his regular tramp to and fro without using them. The lieutent seized him by the collar and shook him roughly, when he instantly awoke, and brought his musket to a ready in proper military style. He was court-martialed for neglect of duty, but acquitted on the ground of somnambulism. He said he was dreaming, while walking his beat, that he was at his home in Massachusetts.

